# Identification of Novel Prognostic Biomarkers Relevant to Immune Infiltration in Lung Adenocarcinoma

**DOI:** 10.3389/fgene.2022.863796

**Published:** 2022-04-27

**Authors:** Zhi Xia, Xueyao Rong, Ziyu Dai, Dongbo Zhou

**Affiliations:** ^1^ Department of Geriatrics, Xiangya Hospital of Central South University, Changsha, China; ^2^ Xiangya Lung Cancer Center, Xiangya Hospital, Central South University, Changsha, China

**Keywords:** lung adenocarcinoma, prognostic biomarkers, immune response, PD-L1, immune infiltration

## Abstract

**Background:** Programmed death ligand-1 (PD-L1) is a biomarker for assessing the immune microenvironment, prognosis, and response to immune checkpoint inhibitors in the clinical treatment of lung adenocarcinoma (LUAD), but it does not work for all patients. This study aims to discover alternative biomarkers.

**Methods:** Public data were obtained from The Cancer Genome Atlas (TCGA) and Gene Expression Omnibus (GEO). Weighted gene co-expression network analysis (WGCNA) and gene ontology (GO) were used to determine the gene modules relevant to tumor immunity. Protein–protein interaction (PPI) network and GO semantic similarity analyses were applied to identify the module hub genes with functional similarities to PD-L1, and we assessed their correlations with immune infiltration, patient prognosis, and immunotherapy response. Immunohistochemistry (IHC) and hematoxylin and eosin (H&E) staining were used to validate the outcome at the protein level.

**Results:** We identified an immune response–related module, and two hub genes (PSTPIP1 and PILRA) were selected as potential biomarkers with functional similarities to PD-L1. High expression levels of PSTPIP1 and PILRA were associated with longer overall survival and rich immune infiltration in LUAD patients, and both were significantly high in patients who responded to anti–PD-L1 treatment. Compared to PD-L1–negative LUAD tissues, the protein levels of PSTPIP1 and PILRA were relatively increased in the PD-L1–positive tissues, and the expression of PSTPIP1 and PILRA positively correlated with the tumor-infiltrating lymphocytes.

**Conclusion:** We identified PSTPIP1 and PILRA as prognostic biomarkers relevant to immune infiltration in LUAD, and both are associated with the response to anti–PD-L1 treatment.

## Introduction

Lung cancer remains the leading cause of cancer death (Travis, 2011). Lung adenocarcinoma (LUAD) is a predominant subtype of lung cancer, and the majority of LUAD patients are diagnosed at an advanced stage, losing the opportunity for surgery (Siegel et al., 2017). Although chemotherapy and targeted therapy can bring survival benefits to advanced patients, drug resistance is inevitable (Molina et al., 2008). With the rapid development of immunotherapy, programmed cell death 1 (PD1) and its ligand (PD-L1) checkpoint inhibitors have become alternative options for advanced patients, enhancing the anticancer immune response by relaunching T-cell–mediated tumor cell death programs through blocking the interaction between PD1 and PD-L1 (Reck, 2018; Dhillon and Syed, 2019). Both the protein and mRNA of PD-L1 can be used to evaluate the tumor immunophenotype, and a high expression of PD-L1 generally predicts benefits from anti–PD1/PD-L1 therapy, resulting in a better prognosis (Conroy et al., 2019).

Although PD-L1 is a well-validated biomarker for immunotherapy response (Shukuya and Carbone, 2016), its positivity does not indicate a certain response to immune checkpoint inhibitors (ICIs), with the objective response rates (ORRs) fluctuating widely (20%–40%) in PD-L1–positive patients. Meanwhile, a subset of PD-L1–negative patients can acquire a good response (Topalian et al., 2012; Brahmer et al., 2015; Garon et al., 2015; Rizvi et al., 2015; Brahmer et al., 2017; Zhang et al., 2020), suggesting the unstable predictive efficiency of PD-L1. Heterogeneity originating from distinct sub-clonal populations of cells could be an important reason for this, with LUAD showing high heterogeneity in immune molecules. PD-L1 expression is diverse among different tumoral regions, such as primary tumors and metastases, so it is likely that immunohistochemistry fails to assess the true PD-L1 status (Ilie et al., 2016; McLaughlin et al., 2016), thus leading to suboptimal decision-making in clinical treatment. Therefore, calculating the immunophenotype from PD-L1 is oversimple, and several studies have confirmed that the signatures related to intra-tumor immune infiltration can effectively predict the response to immunotherapy (Teng et al., 2015; Ock et al., 2016). The aim of this study was to discover the additional immune response–related biomarkers.

Similar to LUAD, skin cutaneous melanoma (SKCM) has a high ORR in first-line immunotherapy (Brahmer et al., 2015; Garon et al., 2015; Larkin et al., 2015), and a systematic review revealed that both PD-L1–negative and PD-L1–positive patients can benefit from the ICIs (Teng et al., 2018), implying the strong immunogenicity of SKCM. Many publications have indicated shared immune characteristics between LUAD and SKCM, which could effectively influence the immune response. A certain proportion of SKCM and LUAD patients possess a similar immune microenvironment, characterized by a high number of mutations or neoantigens, which benefits the patients in anti–PD-L1/PD1 treatment (Chen et al., 2017). In addition, similar intra-tumor heterogeneity and a high leukocyte fraction between SKCM and LUAD have been confirmed. Heterogeneity is associated with the level of tumor-infiltrating immune cells, while tumor types with high leukocyte fractions are generally the most responsive to ICIs (Morris et al., 2016; Thorsson et al., 2018). These transcriptome-based studies provide evidence for the common immunophenotypic basis between LUAD and SKCM, which indicates that they probably have a wide universality in immune-related biomarkers and clinical evaluation. Given that the preserved pattern of the gene module can convey a similar phenotype (Gustafsson et al., 2014), we presume that there could be core modules related to tumor immunity in SKCM and LUAD, and module hub genes, with functional similarities to PD-L1, might be used as biomarkers to evaluate the immunophenotype. Therefore, the introduction of SKCM to identify the common gene module would enable us to reduce thousands of candidate genes to a small number in specific modules, and we can also verify the prognostic or diagnostic value of the potential genes in both SKCM and LUAD based on their common immunophenotypic basis.

**FIGURE 1:**
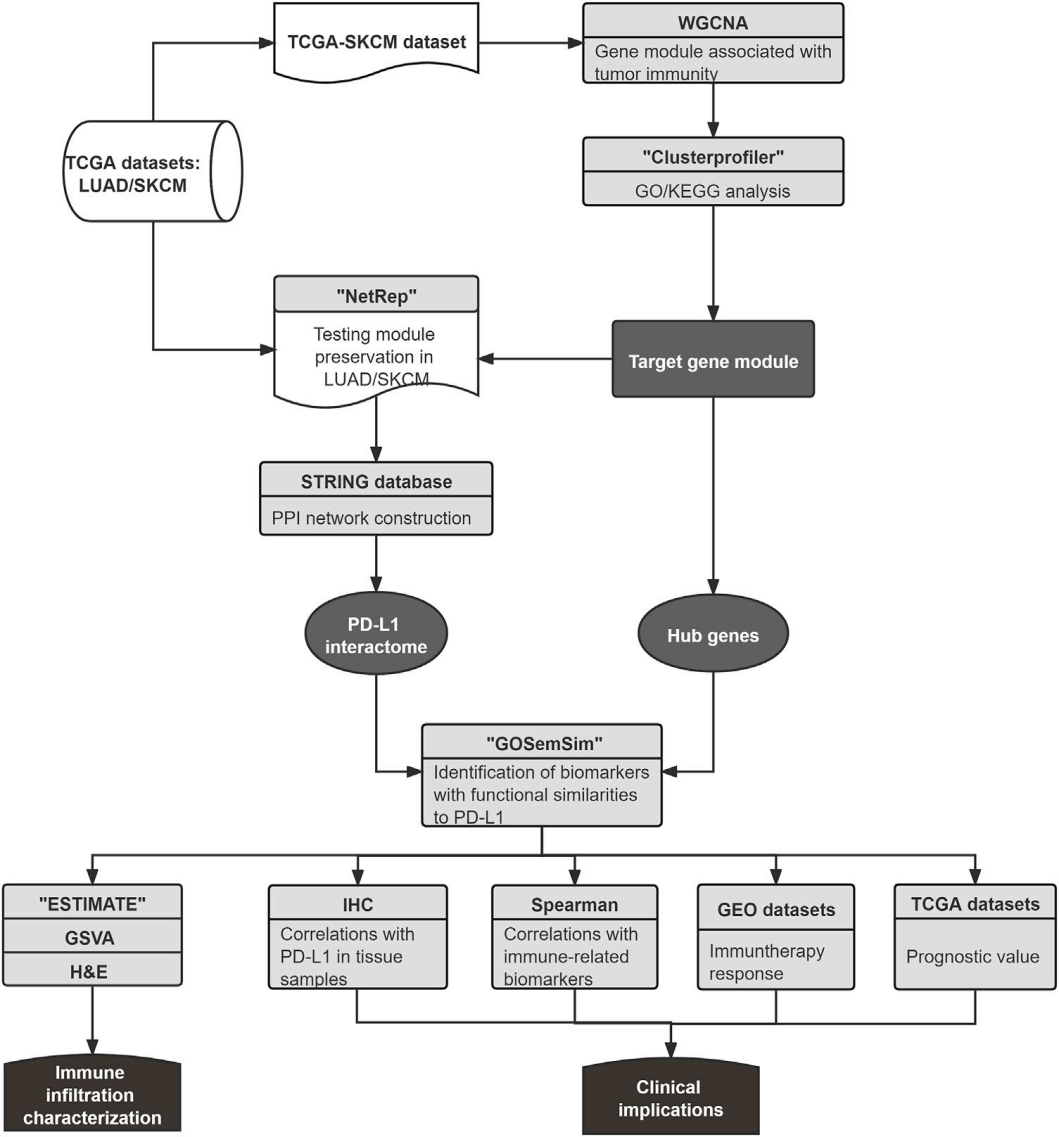
The workflow of this study. First, the shared gene module related to immune regulation of LUAD and SKCM was identified. Next, the PPI network of the gene module was constructed by utilizing the STRING database, and the PD-L1 association network was applied as the PD-L1 interactome, which was used to evaluate the potential biomarkers from the hub genes with functional similarities to PD-L1. Finally, biomarker correlations with patient prognosis, immune biomarkers, immune infiltration, and anti–PD-L1 treatment response were investigated using various approaches.

The pipeline is illustrated in Figure 1. With RNA expression profiles from The Cancer Genome Atlas (TCGA) and Gene Expression Omnibus (GEO), the present study identified that proline–serine–threonine phosphatase–interacting protein 1 (PSTPIP1) and paired Ig-like type 2 receptor alpha (PILRA) have functional similarities to PD-L1, and both are prognostic biomarkers relevant to immune infiltration and the anti–PD-L1 treatment response.

## Materials and Methods

### Data and Clinical Sample Acquisition

The RNA-sequencing (RNA-seq) data [by expectation maximization (Li and Dewey, 2011), RSEM] were obtained from the TCGA (http://cancergenome.nih.gov) by utilizing cBioPortal (Cerami et al., 2012), namely, SKCM (104 samples of primary solid tumors and 368 samples of metastatic tumors) and LUAD (515 samples of primary solid tumors). Two validation data sets were downloaded from GEO (https://www.ncbi.nlm.nih.gov/geo/), meeting the following criteria: 1) data sets with whole transcriptome data, including RNA microarray or sequence data; 2) data sets with human specimens or tissue samples from animal models; 3) data sets with complete information about the technology, platform, and data processing; 4) data sets with available information about the response to anti–PD-L1/PD1 treatment; and 5) the data sets published within 10 years. We used GSE111414 (the RNA-seq data of CD8^+^ peripheral blood lymphocytes (PBLs) from LUAD patients treated with nivolumab) and GSE172320 (the RNA microarray data of samples from SKCM mice treated with anti–PD-L1) to determine the implications of PSTPIP1 and PILRA in the anti–PD-L1 treatment response. In addition, GSE68571 (the RNA microarray data of 436 LUAD samples with available differentiation information) was downloaded to explore the association between PSTPIP1/PILRA and LUAD differentiation. Data normalization was performed using the R package “limma.”

The LUAD pathological section materials of 18 patients, that is, 9 PD-L1–positive samples and 9 PD-L1–negative samples, were acquired from the Department of Pathology, Xiangya Hospital of Central South University, and PD-L1 expression was confirmed by pathologists using the PD-L1 immunohistochemistry (IHC) 22C3 pharmDx assay (Hirsch et al., 2017). The clinicopathological characteristics of the patients are listed in Supplementary Table S1. The present study was approved by the ethics committee of Xiangya Hospital of Central South University.

### Weighted Gene Co-Expression Network Analysis

WGCNA was performed using the R package “WGCNA” (Langfelder and Horvath, 2008). Modules produced by WGCNA, named by different colors, refer to genes that share a similar connectivity pattern. Module membership (MM) is the relevance of the expression profile to each module eigengene. Hub genes, the central point of the gene module architecture, were defined as those genes with MM > 0.8. The R package “NetRep” was used to evaluate the replication and preservation of the target module from seven module preservation statistics (Ritchie et al., 2016). According to the tutorial, a gene module was considered strongly preserved if the *p* value was <0.01 for all preservation statistics, weakly preserved if the *p* value was <0.01 for one or more, but not all, test statistics, and no evidence if no test statistics had a *p* value < 0.01.

### Gene Ontology and Pathway Enrichment Analysis

GlueGO (Bindea et al., 2009) and the R package “Clusterprofiler” (Yu et al., 2012) were applied to the Gene Ontology (GO) and Kyoto Encyclopedia of Genes and Genomes (KEGG) pathway analysis and outcome visualization. Based on similarly associated genes, GO parent–child terms construct a hierarchy from the global to the specific level. Significant GO terms are summarized into representative terms by the fusion of the GO parent–child terms.

### Protein–Protein Interaction Network Construction

The PPI information of the core module was obtained from the STRING database (https://string-db.org/), which offers the most confident interactions among module genes. We constructed a PPI network by using the Cytoscape 3.4.0 software (Shannon et al., 2003). Subsequently, the plug-in Molecular Complex Detection (Bader and Hogue, 2003) and GO analysis were applied to determine the central submodule related to tumor immunity, which helps to identify the core of the immune-related network in the target module (with the parameters: degree cutoff = 2, K-core = 2, and node score cutoff = 0.2).

### Gene Ontology Semantic Similarity Analysis

The assessment of GO semantic similarity between genes can predict their relevant functions (Tedder et al., 2010). Based on the PPI information, the protein-coding genes having a connection with PD-L1 were incorporated into the PD-L1 interactome. Using the function “mgeneSim” in the R package “GOSemSim” (Yu et al., 2010), the semantic similarities between each hub gene and the PD-L1 interactome were calculated by taking the molecular function (MF) and cellular component (CC) of the GO topological structure into account. The Wang method was used in this process, which can accurately determine the semantic similarities of genes *via* a graph-based strategy (Wang et al., 2007). We used the geometric mean of semantic similarities in MF and CC to score the functional correlations between each hub gene and the PD-L1 interaction partners. A hub gene with a high score was generally considered to have a high probability of functional similarity to PD-L1, meaning it could be implicated in tumor immune regulation and relevant to the PD-L1 association network. We ranked hub genes by their average functional similarity score, providing an initial evaluation for their functional similarities to PD-L1.

### Survival Analysis

Survival analysis was performed using the R packages “survminer” and “survival.” Based on the mRNA expression of biomarkers, the samples were divided into two groups to plot Kaplan–Meier survival curves. A high expression was defined as samples with biomarker expression values above the median value, whereas a low expression was defined as samples with biomarker expression values below the median value.

### Immune Infiltration Characterization

The R package “ESTIMATE” was used to quantify the total levels of tumor-infiltrating immune cells. Based on the unique properties of the transcriptional profiles, “ESTIMATE” performs a single sample gene set enrichment algorithm, which calculates the strength of the concerted behavior of the immune-related gene sets in each tumor sample (Yoshihara et al., 2013). We further utilized the R package “GSVA” to calculate the enrichment score of each infiltrating lymphocyte. By implementing a nonparametric unsupervised method to score the gene set enrichment in the gene microarray and RNA-seq data, “GSVA” transforms the data from a gene to a gene set by the sample matrix, allowing for the calculation of an enrichment score for each sample without information about explicitly modeling phenotypes (Hänzelmann et al., 2013). According to the median expression value of biomarkers, samples were separated into high expression and low expression groups, and we explored the status of tumor-infiltrating lymphocytes (TILs) in each tissue sample. A list of immune metagenes whose expressions have been shown to accurately predict the infiltration of immune cell populations was utilized as an input object for the “GSVA” (Angelova et al., 2015).

### Assay Methods

IHC was used to determine the protein levels of PSTPIP1 and PILRA. LUAD tissue samples were sectioned into 4-mm-thick slices, deparaffinized in xylene and rehydrated in a series of graded alcohols. Antigen retrieval was performed by immersing the slides in sodium citrate. Endogenous peroxidase was blocked by a 10-min incubation with 3% H_2_O_2_. Next, the slices were incubated with the primary antibodies anti-PSTPIP1 (11951-1-AP, rabbit, polyclonal, dilution 1:50, Proteintech, Wuhan, China), anti-PILRA (orb38981, rabbit, polyclonal, dilution 1:200, Biorbyt, Cambridge, United Kingdom), and PBS (blank control) overnight at 4°C, washed three times with PBS, and incubated with a horseradish peroxidase (HRP)–conjugated secondary antibody (ab205718, Abcam, Cambridge, United Kingdom) for 30 min. Finally, immunostaining was performed with a diaminobenzidine substrate kit (ab64238, Abcam, Cambridge, United Kingdom). According to the outcome of IHC, we equally separated LUAD samples into high- and low-expression groups, and TILs were calculated in hematoxylin and eosin (H&E)–stained sections according to the standardized evaluation of TILs in breast cancer (Salgado et al., 2015). The IHC and H&E staining results were evaluated using the ImageJ software. Three to five typical fields of view per image were measured, and we obtained the mean value. The average optical density (AOD) or stromal TILs were calculated by counting the average of three pathological sections from each sample. The median values of the AOD expressed in each sample were used as the cutoff value, and the samples were divided into the high- or low-expression group.

### Statistical Analysis

R statistical software (v.3.6.1) was used for statistical analyses and graphical visualization. The analysis was performed on log2-transformed values. Spearman’s correlation test was applied to assess the relationships among biomarkers. The Wilcoxon test was used to compare the distributions of two sets of any continuous variable. The Kruskal–Wallis test was used to compare the distributions of three or more sets of any continuous variable. Null hypotheses were rejected at a two-sided *p* value lower than 0.05, unless otherwise indicated.

## Results

### Identification of the Gene Module Related to Tumor Immunity

**FIGURE 2:**
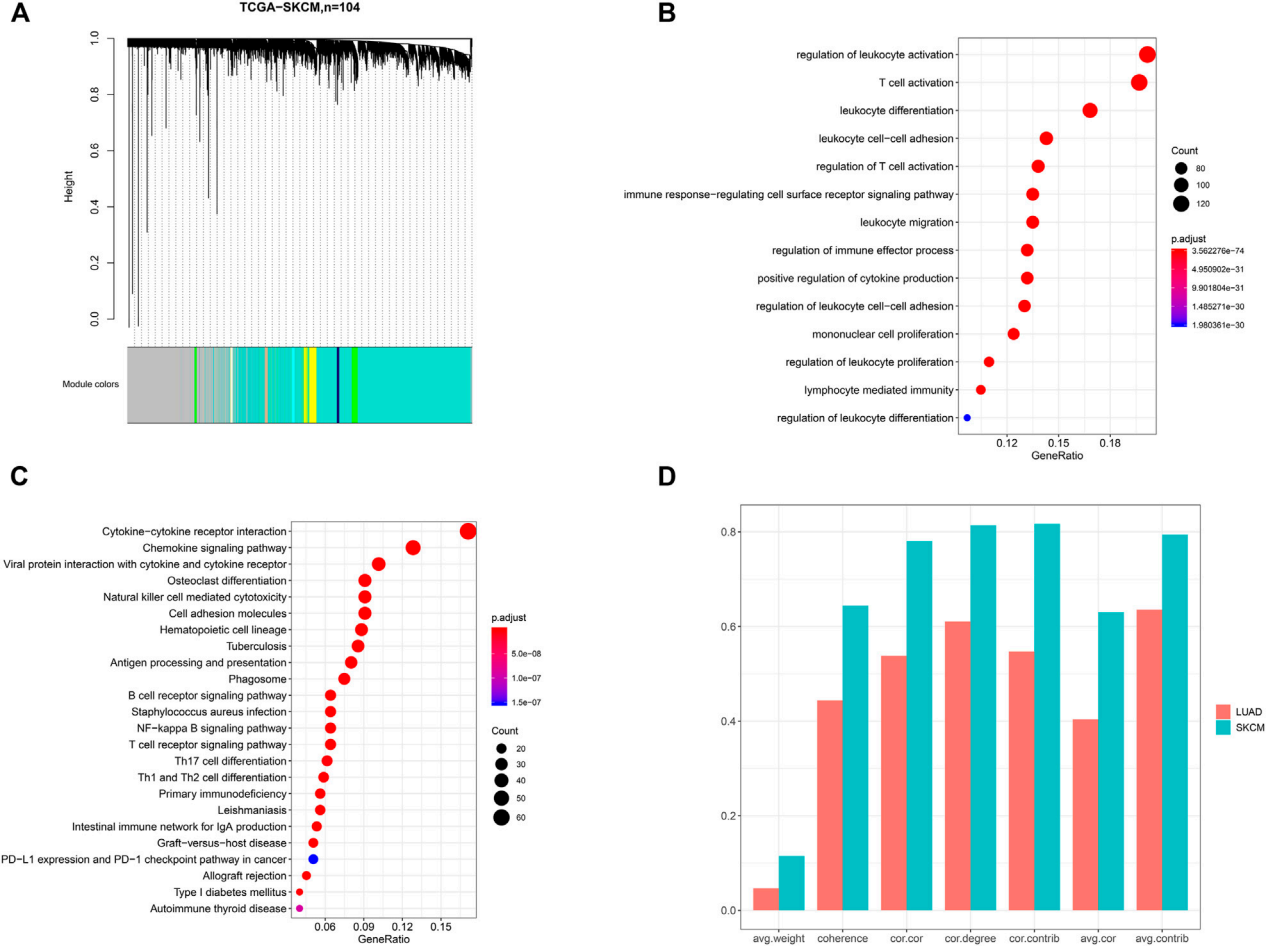
Identification of a gene module associated with tumor immunity. **(A)** Dendrogram of 104 skin cutaneous melanoma (SKCM) samples. The results of **(B)** GO and **(C)** KEGG pathway analyses for the genes belonging to the brown module. **(D)** Assessment of the preservation pattern of the brown module in SKCM and lung adenocarcinomas (LUAD) from seven module preservation statistics, and the bar plots showing the observed value of each module preservation statistic. cor.cor: the concordance of the correlation structure; avg.cor: the average magnitude of the correlation coefficients of the module; avg.weight: the average magnitude of edge weights; cor.degree: the concordance of the weighted degree of nodes; cor.contrib: the concordance of the node contribution; avg.contrib: the average magnitude of the node contribution; coherence: the proportion of variance in the module data explained by the module’s summary profile vector.

To identify the core module, we first performed WGCNA on 104 SKCM samples from primary lesions. The weighted gene co-expression network identified 21 modules (Figure 2A). According to the results of the GO and KEGG analyses, we identified the brown module, which consists of 743 protein-coding genes such as PD-L1, involved in tumor immune regulation. The bubble diagram shows the enriched GO terms implicated in the functional regulation of multiple lymphocytes and immune-related processes (Figure 2B). KEGG analysis revealed that immune cell–mediated and PD-L1–related pathways were enriched, including the “B-cell receptor signaling pathway,” “T-cell receptor signaling pathway,” and “PD-L1 expression and PD-1 checkpoint pathway in cancer,” which are closely related to tumor immunity and immunotherapy response (Figure 2C). Compared to other modules, GO terms associated with immune-related regulation and biological processes were almost concentrated in the brown module, meaning that the module is likely to be in charge of tumor immunity, and thus it is a candidate for the subsequent analysis. Then, we calculated the module’s preservation pattern, showing its strong preservation in LUAD and metastatic SKCM, which means that this gene module could be related to the common immunophenotype in SKCM and LUAD (Figure 2D).

### Construction of the Protein–Protein Interaction Network

**FIGURE 3:**
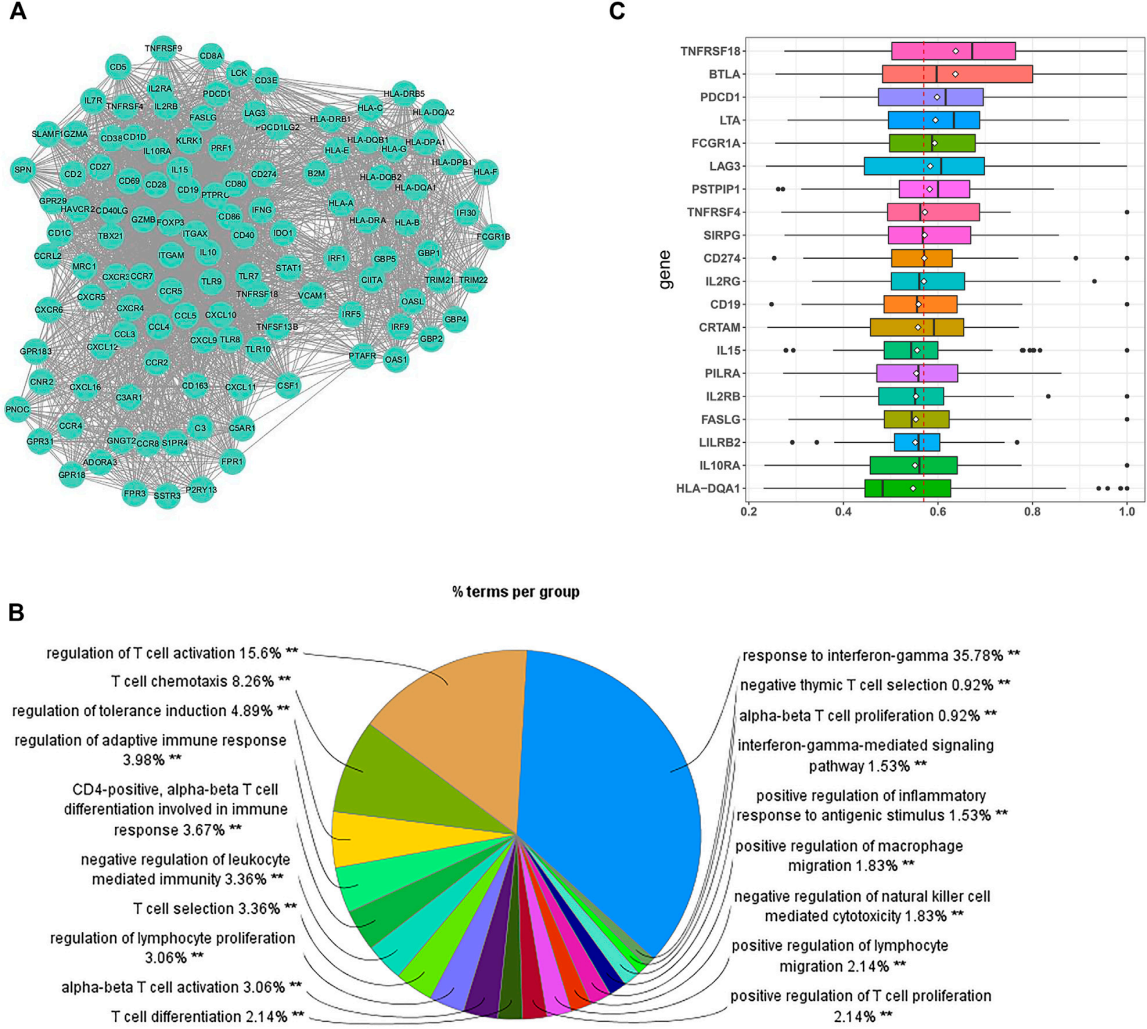
Identification of the potential biomarkers relevant to tumor immunity. **(A)** The submodule of the PPI network involved in immune regulation. **(B)** GO analysis for the genes in the submodule. Each section of the pie chart shows the representative GO global terms. The size of each section is associated with the percent of genes within the submodule. **(C)** Summary of the functional similarities for the top 20 protein-coding genes in the PD-L1 (CD274) interactome. The distribution of functional similarity scores was summarized as boxplots. The lines and rhombuses in the boxes indicate the mean and median of the functional similarity scores, respectively. The dashed line represents the median value of PD-L1. **p < 0.01.

A total of 199 hub genes were identified in the brown module. Then, we screened the central submodule in the brown module by constructing the PPI network, and a submodule composed of 119 genes was significantly associated with tumor immunity (Figure 3A). In addition to regulating the functions of multiple lymphocytes, most protein-coding genes in the submodule are involved in “T-cell activation” and “response to interferon-gamma (IFN-γ)” (Figure 3B). Based on the connections among protein-coding genes provided by the STRING database, we applied the PD-L1 association network as the PD-L1 interactome, which is composed of 83 protein-coding genes, including PD-L1 and its regulators. We utilized the R package “GOSemSim” to score functional similarities between the PD-L1 interactome and 199 hub genes. Genes with high scores are likely to have similar molecular functions to PD-L1. According to the results, we ranked the hub genes by the average functional similarity scores (Figure 3C) and found that PD-L1 (namely, CD274, a hub gene of the brown module) had the 10th highest average score among the hub genes, and the average score of the first was significantly higher than that of PD-L1 (*p* = 0.0019). No significant differences were found between the average scores of PD-L1 and the genes ranked second to 19th (*p* > 0.05), while the average score of the genes ranked below the 19th was significantly lower than that of PD-L1 (*p* < 0.05), meaning that the genes ranked second to 19th are most likely to play a similar role to PD-L1 in MF and CC. Except for PSTPIP1 and PILRA, the other genes with high scores have been identified to have functionally relevant roles in the immune checkpoint, tumoral immune cells, and immune infiltration, while few studies have reported the role of PSTPIP1 and PILRA in tumor immunity.

### Implications in Tumor Immunity

**FIGURE 4:**
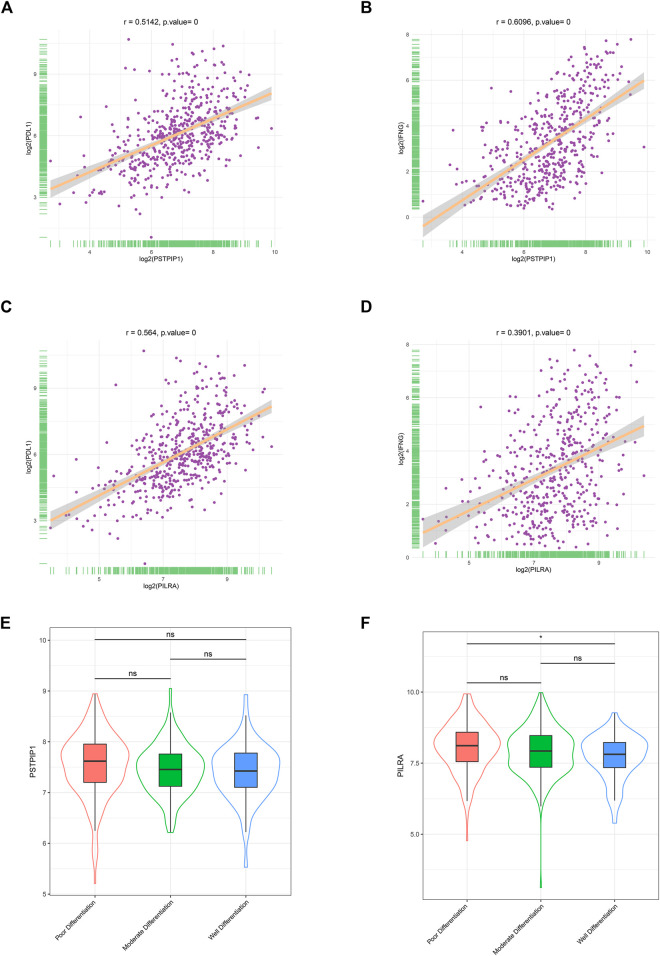
The correlations between PSTPIP1/PILRA and immune biomarkers in LUAD. Scatter plots showing the correlation between **(A)** PSTPIP1 and PD-L1, **(B)** PSTPIP1 and IFN-γ, **(C)** PILRA and PD-L1, and **(D)** PILRA and IFN-γ in the LUAD samples (*n* = 515). Violin plots showing the expression of **(E)** PSTPIP1 and **(F)** PILRA in the LUAD samples with different degrees of differentiation (including 167 with poor differentiation, 209 with moderate differentiation, and 60 with well differentiation). ns indicates *p* ≥ 0.05, *p < 0.05.

We calculated the relationship between PSTPIP1/PILRA and the immune biomarkers, namely, PD-L1 and IFN-γ. We observed a significantly positive correlation between PSTPIP1/PILRA and PD-L1/IFN-γ in the LUAD samples (Figures 4A–D), and similar outcomes were observed in SKCM (Supplementary Figure S1). Given that LUAD differentiation can influence the expression of PD-L1 (Takada et al., 2016), we further explored the correlation between PSTPIP1/PILRA and tumor differentiation. The results revealed that LUAD tissue samples with distinct differentiation had similar levels of PSTPIP1. Compared with poorly differentiated samples, PILRA decreased in well-differentiated LUAD, but no significant difference was observed between the moderately and well-differentiated samples or between the poorly and moderately differentiated samples (Figures 4E,F).

### Immune Infiltration and Survival Analysis

**FIGURE 5:**
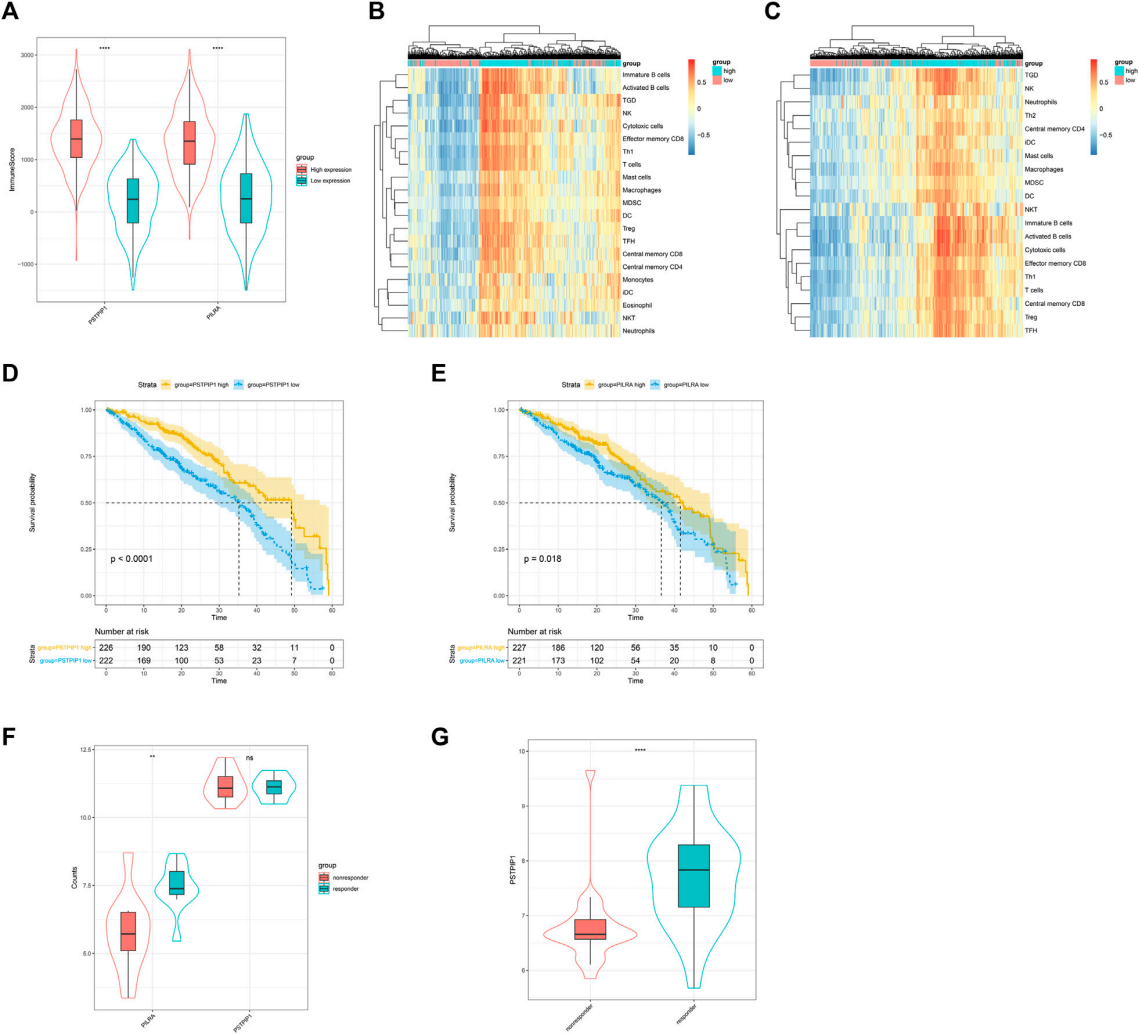
The influence of PSTPIP1 and PILRA on immune infiltration and prognosis in LUAD. Violin plots showing **(A)** the immune score in samples with low or high expression of PSTPIP1 and PILRA. GSVA-derived clustering heat maps of differentially infiltrated immune cell populations between the high and low expression groups of **(B)** PSTPIP1 and **(C)** PILRA. Only lymphocytes with log(fold change) > 0.2 are shown. The influence of **(D)** PSTPIP1 and **(E)** PILRA on the overall survival time of LUAD patients. The yellow line indicates samples with highly expressed genes and the blue line indicates samples with lowly expressed genes. Violin plots showing **(F)** the expression of PSTPIP1 and PILRA in the LUAD patients with different responses to nivolumab (including five responders and five nonresponders) and **(G)** the expression of PSTPIP1 in SKCM mice with different responses to anti–PD-L1 treatment (including 27 responders and 23 nonresponders). ns indicates *p* ≥ 0.05, **p < 0.01, ****p < 0.0001.

We explored the correlation between PSTPIP1/PILRA and TILs. The ESTIMATE immune scores revealed that the LUAD samples with a high expression of PSTPIP1 and PILRA had significantly richer immune infiltration (Figure 5A). GSVA confirmed that the high expression of PSTPIP1 and PILRA led to an increased enrichment of multiple lymphocytes (Figures 5B,C). We next investigated the prognostic value of PSTPIP1 and PILRA and found that the high expression of PSTPIP1 and PILRA contributed to longer overall survival (OS) (Figures 5D,E). Similar results were confirmed in SKCM (Supplementary Figure S2A–E). We investigated the correlations between PSTPIP1/PILRA and the anti–PD-L1 treatment response in LUAD patients and found that PILRA mRNA was significantly high in CD8^+^ PBLs from the patients who responded to nivolumab, but the expression of PSTPIP1 was not different between the responders and nonresponders (Figure 5F). However, the significantly increased expression of the PSTPIP1 homologous gene was confirmed in tumor tissues from the SKCM mice that responded to the anti–PD-L1 treatment (Figure 5G).

### Correlation Between Tumor-Infiltrating Lymphocytes and Biomarkers at the Protein Level

**FIGURE 6:**
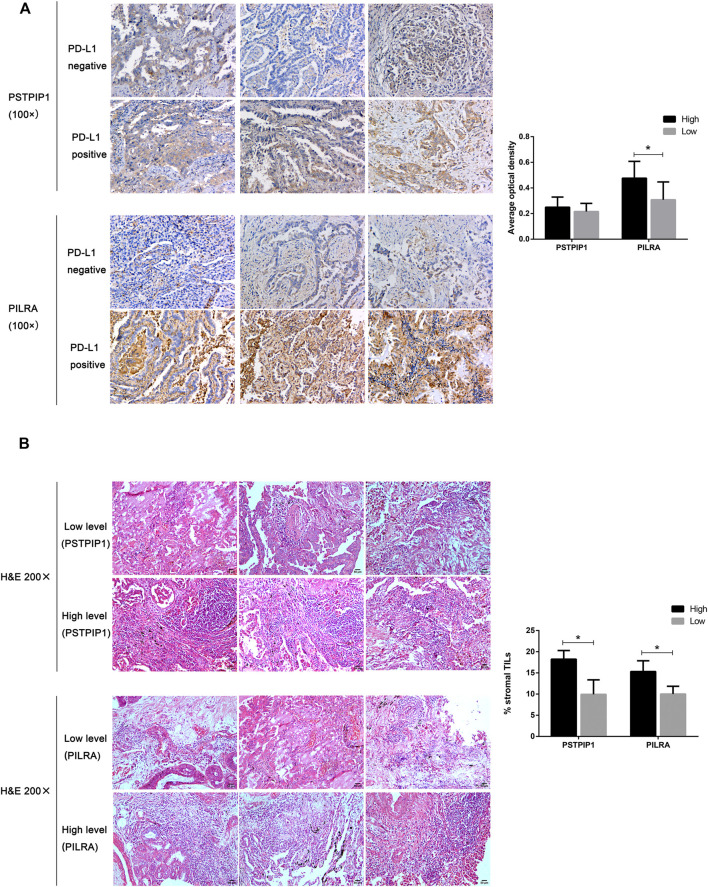
Histopathological examples of PSTPIP1/PILRA expression and tumor-infiltrating lymphocytes (TILs) in LUAD. **(A)** PSTPIP1 and PILRA expression is demonstrated by brown staining, and the bar plot shows the average optical density of PSTPIP1 and PILRA in the PD-L1–positive or PD-L1–negative LUAD tissue samples. **(B)** The TILs are displayed as purple spots in hematoxylin and eosin staining, and bar plots show the stromal TILs in tissue samples with high and low PSTPIP1/PILRA expressions. *p < 0.05.

We validated the protein expression of PSTPIP1 and PILRA in the PD-L1–positive and PD-L1–negative LUAD tissue samples. In contrast to the PD-L1–negative samples, we observed that the protein level of PILRA was higher in the PD-L1–positive samples (*p* = 0.0174) (Figure 6A). Although PSTPIP1 showed a relatively higher expression in the PD-L1–positive tissues, there was no statistical significance when compared to the PD-L1–negative samples (*p* = 0.3355) (Figure 6A). At the same time, both PSTPIP1 and PILRA high expression samples possessed relatively richer TILs, while relatively lower TILs were prone to exist in samples with low PSTPIP1 and PILRA expressions (*p* < 0.05), and stromal TILs tended to exceed 10% in the LUAD samples with a high expression of PSTPIP1 and PILRA (Figure 6B).

## Discussion

This study applied network analysis methods to transcriptome data to explore the immune basis of LUAD and identified that it shares a strongly preserved immune response–related module with SKCM. The hub genes PSTPIP1 and PILRA are novel prognostic biomarkers positively correlated with TILs, and both could be used to predict immunotherapy response.

TILs are well-validated factors influencing the ICI response (Gibney et al., 2016), and PD-L1–positive SKCM accompanied by high TILs accounts for 40% of cases, which is the favorable immunophenotype for immunotherapy response (Teng et al., 2015). This provides the basis for SKCM patients with an ideal clinical efficacy in various immunotherapeutic approaches (Tsai et al., 2014). Researchers found that some LUAD cases have an immunophenotype similar to that of SKCM (Morris et al., 2016; Chen et al., 2017; Thorsson et al., 2018), which could be explained by the existence of a shared gene module related to tumor immunity. In the present study, we identified this sharing module, and two hub genes were considered to have functional similarities to PD-L1. The PSTPIP1 hub gene was related to T-cell activation, differentiation, and migration, modulating the function of innate immune cells and the innate immune response, and its mutation was confirmed as a crucial driver of immunodeficiency and auto-inflammatory diseases (Holzinger and Roth, 2016; Janssen et al., 2018). The PILRA hub gene was primarily expressed on multiple immune cells (Kogure et al., 2011; Sun et al., 2012), which can trigger increased natural killer cell–mediated IFN-γ secretion by binding to o-glycosylated receptors (Ophir et al., 2016). However, few studies have reported the correlation between these two protein-coding genes and the tumor-related immune response, and we confirmed that they have a relationship with multiple infiltrating lymphocytes and tumor immune regulation.

The majority of module genes are involved in the response to IFN-γ, a well-established biomarker for tumor immunity (Dong et al., 2016). IFN-γ is secreted from the TILs, which compromises antitumor immunity by promoting PD-L1 expression on the surface of tumor and immune cells (Bald et al., 2014; Remon et al., 2016; Ayers et al., 2017). Previous studies have reported a positive association between IFN-γ and PD-L1 at the mRNA level (Hayano et al., 2017), and a high expression of tumoral IFN-γ mRNA was associated with a good response to the PD-L1 inhibitor durvalumab in non–small-cell lung cancer (NSCLC) patients; the ORR in IFN-γ–positive patients reached 33%, while it was 8% in IFN-γ–negative patients, and the highest ORR (46%) was observed in cases with a coexisting positive expression of IFN-γ and PD-L1 (Higgs et al., 2015). Both PSTPIP1 and PILRA were positively associated with PD-L1 and IFN-γ, supporting their influence on the immune response. PILRA was lower in well-differentiated LUAD, which contradicts the widely validated correlations between the high PD-L1 expression and good differentiation. Therefore, to some extent, we can exclude the possibility that PSTPIP1 and PILRA levels are correlated with tumor differentiation rather than immune biomarkers. However, the protein expression of PSTPIP1 was not significantly increased in the PD-L1–positive LUAD tissues, implying that its correlation with PD-L1 might be indirect.

The TILs (mainly CD8^+^ T cells) are important biomarkers for assessing the immune microenvironment, and both the tumor cell surface PD-L1 and intratumoral IFN-γ are associated with the level of the TILs (Dong et al., 2016; Tang et al., 2016). TIL-derived IFN-γ induces the expression of PD-L1, which in turn suppresses TIL-mediated antitumor immunity (Gowrishankar et al., 2015). We confirmed that TILs were significant in samples with high PSTPIP1 and PILRA levels. Tumors positive for PD-L1 and TILs are adaptively resistant to elimination by TILs, and this immunophenotype is most likely to respond to anti–PD-L1 therapy (Zhang and Chen, 2016). We found that the LUAD patients who responded to PD-1/PD-L1 blockade treatment tended to have a high expression of PILRA mRNA in CD8^+^ PBLs. Although PSTPIP1 expression showed no significant difference, we confirmed that significantly increased PSTPIP1 expression was observed in tumor tissues from SKCM mice that responded to anti–PD-L1 treatment. This difference might be derived from a distinctive gene repertoire between CD8^+^ TILs and PBLs (Mohme et al., 2018). The high expression of PSTPIP1 inhibits CD3-dependent T-cell activation, which is significantly higher in TILs rather than in PBLs (Marcos et al., 2014; Lukesova et al., 2015), leading to adaptive resistance. Therefore, high expression of PSTPIP1 in the TILs could indicate a clinical response after immunotherapy of relaunching T-cell–mediated actions. We presumed that the differential expression of PSTPIP1 between the responders and non-responders might be observed in TILs rather than PBLs. Previous studies have confirmed that subpopulations of TILs, such as effector memory and central memory CD8^+^ cells, effector memory CD4^+^ cells, natural killer cells, and activated dendritic cells, are associated with good prognosis (Angelova et al., 2015), leading to an improved survival time in NSCLC patients (Thomas et al., 2013; Teng et al., 2015; Teng et al., 2016). The survival analysis is consistent with these conclusions, and the survival benefits of high PSTPIP1 and PILRA expressions are possibly due to rich TILs, and their influence is also in accord with their positive correlations with PD-L1, which confirms that both the protein and mRNA levels of PD-L1 are associated with increased TILs and OS in NSCLC patients (Velcheti et al., 2014; Ma et al., 2020). In general, PSTPIP1 and PILRA act as biomarkers for TILs and thus have positive correlations with PD-L1 and IFN-γ.

In the present study, we confirmed that PSTPIP1 and PILRA have a relationship with the TILs at both the protein and gene levels. However, selection bias was inevitable because of the small sample sizes, which is the main limitation of this study. Moreover, there was a lack of sufficient available data to firmly validate the conclusions made from the TCGA. Further study is needed to verify the clinical value of PSTPIP1 and PILRA in the additional samples and to explore their molecular functions in tumor immune regulation.

## Conclusion

In conclusion, the present study demonstrated that PSTPIP1 and PILRA can reflect the status of TILs and work as prognostic biomarkers, and they could act as biomarkers relevant to the anti–PD-L1 treatment response.

## Data Availability

The original contributions presented in the study are included in the article/Supplementary Material. Further inquiries can be directed to the corresponding author.

## Supplementary Material

Supplementary Figure S1 Correlations between PSTPIP1/PILRA and immune biomarkers in skin cutaneous melanoma (SKCM). Scatter plots showing the correlations between **(A)** PSTPIP1 and PD-L1, **(B)** PSTPIP1 and IFN-γ, **(C)** PILRA and PD-L1, and **(D)** PILRA and IFN-γ in SKCM samples (*n* = 472).

Supplementary Figure S2 The influence of PSTPIP1 and PILRA on immune infiltration and prognosis in SKCM. Violin plots showing **(A)** the immune score in samples with low or high expression of PSTPIP1 and PILRA. GSVA-derived clustering heatmaps of differentially infiltrated immune cell populations between the high and low expression groups of **(B)** PSTPIP1 and **(C)** PILRA. Only lymphocytes with log (fold change) > 0.2 are shown. The influence of **(D)** PSTPIP1 and **(E)** PILRA on the overall survival time of SKCM patients. The yellow line indicates samples with highly expressed genes, and the blue line indicates samples with lowly expressed genes. *****p* < 0.0001.

Supplementary Table S1 Clinical and pathological characteristics of lung adenocarcinomas patients.

## Abbreviations

CC, cellular component; GO, gene ontology; GSVA, gene set variation analysis; H&E, hematoxylin and eosin; IHC, immunohistochemistry; IFN-γ, interferon-gamma; KEGG, Kyoto Encyclopedia of Genes and Genomes; LUAD, lung adenocarcinoma; MF, molecular function; MM, Module membership; NSCLC, non–small-cell lung cancer; ORRs, objective response rates; PBLs, peripheral blood lymphocytes; PD-L1, programmed death ligand-1; PD1, programmed cell death 1; PILRA, paired Ig-like type 2 receptor alpha; PSTPIP1, proline–serine–threonine phosphatase–interacting protein 1; PPI, protein–protein interactions; SKCM, skin cutaneous melanoma; TCGA, The Cancer Genome Atlas; TILs, tumor-infiltrating lymphocytes; WGCNA, weighted co-expression network analysis.
