# Supplementary material for: Identification of Novel Prognostic Biomarkers Relevant to Immune Infiltration in Lung Adenocarcinoma
Source: Front Genet. 2022 Apr 27;13:863796. doi: 10.3389/fgene.2022.863796 (PMC9092026; doi:10.3389/fgene.2022.863796)
Supplement: Supplementary file 1 [file Table1.docx]

Table S1. Clinical and pathological characteristics of lung adenocarcinomas patients

|  |  |  | PD-L1 |  |
| --- | --- | --- | --- | --- |
| Characteristics | Total, No. |  | No. |  |
|  |  | Negative | Positive  (1-50%) | Strong positive  (>50%) |
| Patients | 18 | 9 | 6 | 3 |
| Median age | 58.28 |  |  |  |
| Sex |  |  |  |  |
| Male | 11 | 7 | 3 | 1 |
| Female | 7 | 3 | 3 | 1 |
| Differentiation^&^ |  |  |  |  |
| Poor | 3 | 2 | 0 | 1 |
| Moderate -poor | 4 | 3 | 0 | 1 |
| Moderate | 9 | 4 | 4 | 1 |
| Moderate -well | 0 | 0 | 0 | 0 |
| Well | 2 | 0 | 2 | 0 |

^&^ Tumor differentiation is classified into 5 grades from the poor- to well-differentiated.
