# Supplementary figures and images for: Identification of Novel Prognostic Biomarkers Relevant to Immune Infiltration in Lung Adenocarcinoma
Source: Front Genet. 2022 Apr 27;13:863796. doi: 10.3389/fgene.2022.863796 (PMC9092026; doi:10.3389/fgene.2022.863796)

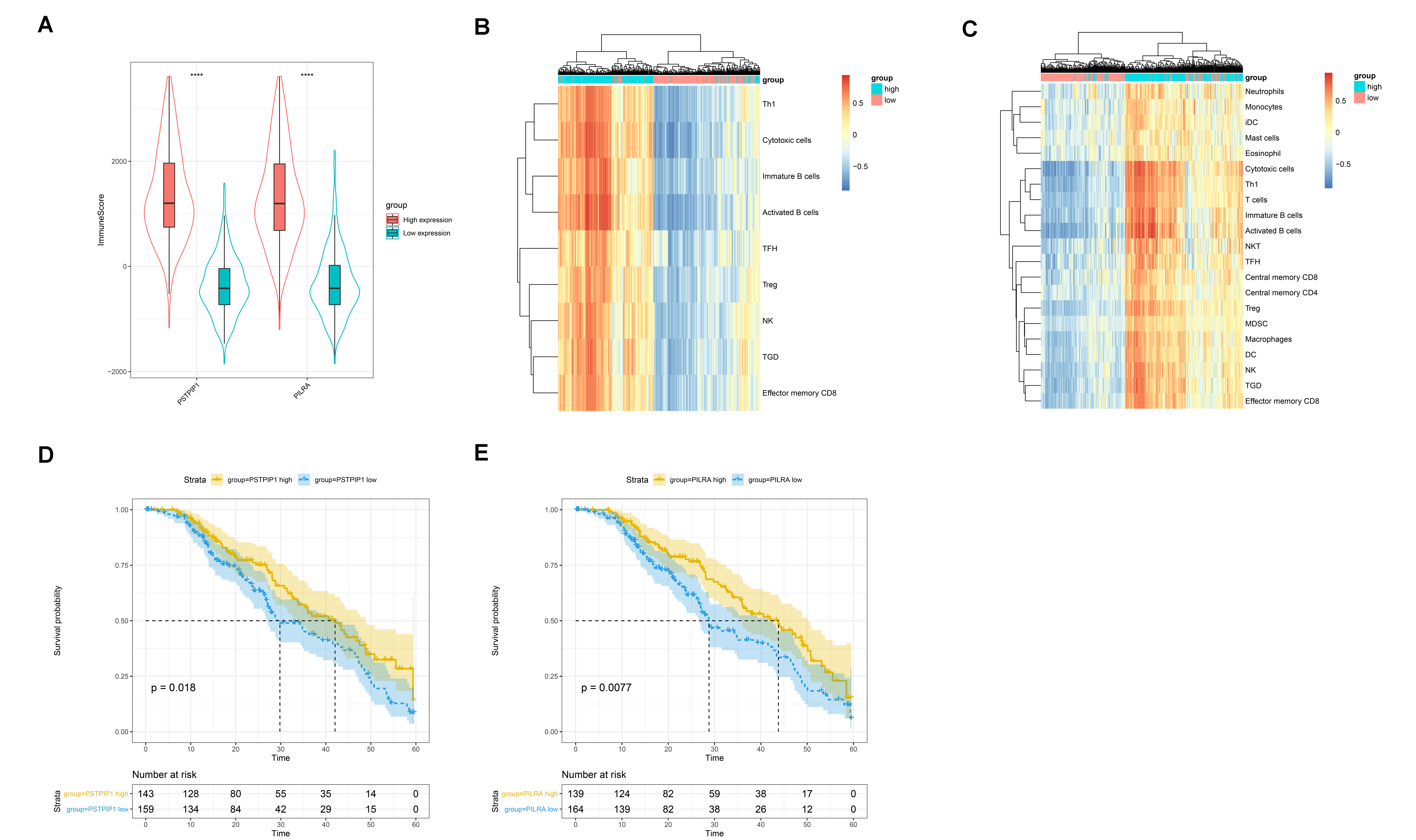

Supplement: Supplementary file 2 [file Image2.TIF]

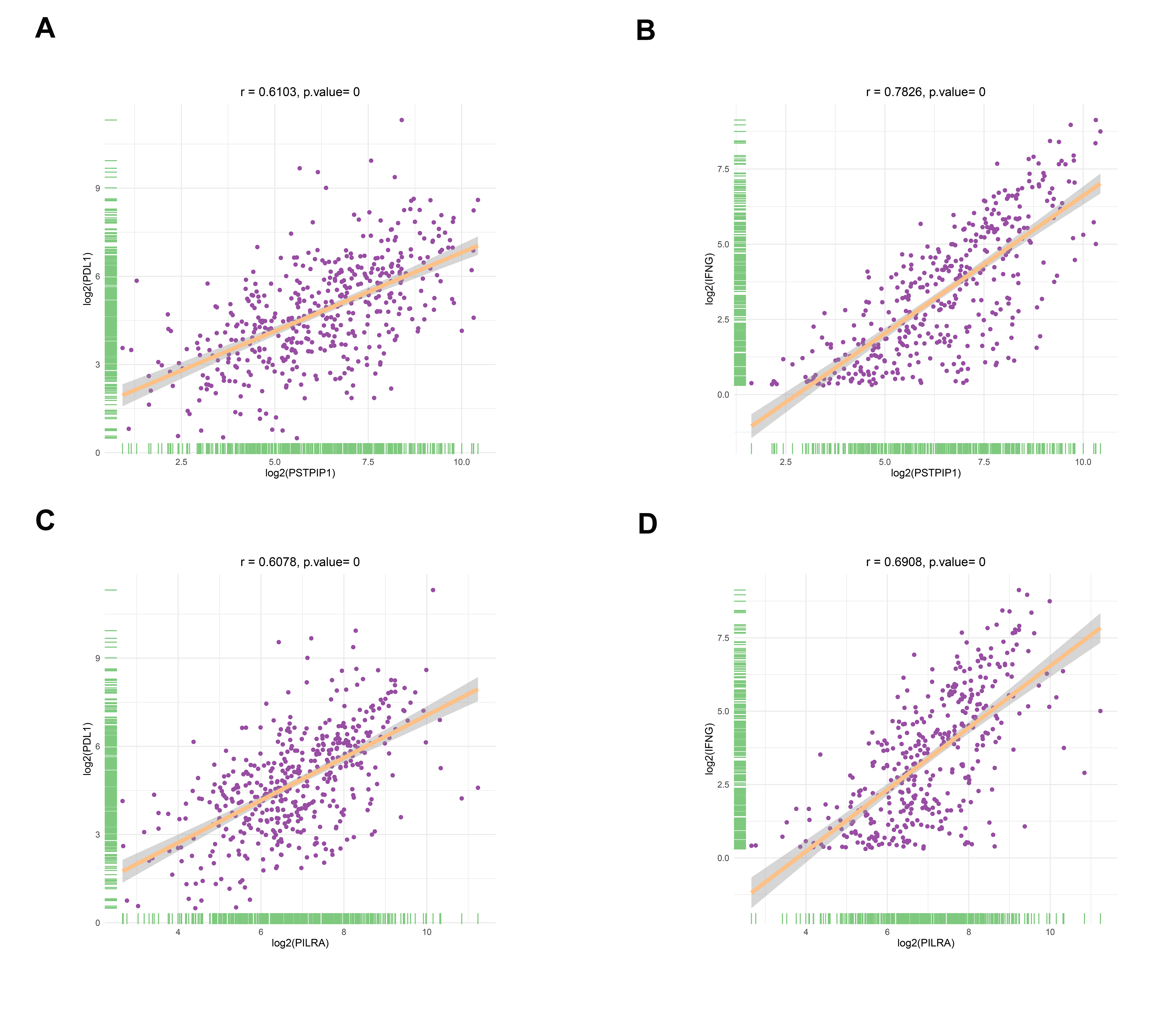

Supplement: Supplementary file 3 [file Image1.TIF]
